# Kaiso Regulates DNA Methylation Homeostasis

**DOI:** 10.3390/ijms22147587

**Published:** 2021-07-15

**Authors:** Darya Kaplun, Alexey Starshin, Fedor Sharko, Kristina Gainova, Galina Filonova, Nadezhda Zhigalova, Alexander Mazur, Egor Prokhortchouk, Svetlana Zhenilo

**Affiliations:** 1 Federal State Institution «Federal Research Centre «Fundamentals of Biotechnology» of the Russian Academy of Sciences», 119071 Moscow, Russia; kaplun.dascha@gmail.com (D.K.); starshin.alexey@gmail.com (A.S.); fedosic@gmail.com (F.S.); filonova112233@gmail.com (G.F.); nzhigalova@gmail.com (N.Z.); mazur.am@gmail.com (A.M.); 2 Institute of Gene Biology RAS, 119071 Moscow, Russia; 3 Centre for Strategic Planning of FMBA of Russia, 119071 Moscow, Russia; gaynovakristina@gmail.com

**Keywords:** DNA methylation, Kaiso, cancer, de novo DNA methyltransferases, DNMT3a/3b

## Abstract

Gain and loss of DNA methylation in cells is a dynamic process that tends to achieve an equilibrium. Many factors are involved in maintaining the balance between DNA methylation and demethylation. Previously, it was shown that methyl-DNA protein Kaiso may attract NCoR, SMRT repressive complexes affecting histone modifications. On the other hand, the deficiency of Kaiso resulted in reduced methylation of ICR in *H19/Igf2* locus and *Oct4* promoter in mouse embryonic fibroblasts. However, nothing is known about how Kaiso influences DNA methylation at the genome level. Here we show that deficiency of Kaiso led to whole-genome hypermethylation, using Kaiso deficient human renal cancer cell line obtained via CRISPR/CAS9 genome editing. However, Kaiso serves to protect genic regions, enhancers, and regions with a low level of histone modifications from demethylation. We detected hypomethylation of binding sites for Oct4 and Nanog in Kaiso deficient cells. Kaiso immunoprecipitated with de novo DNA methyltransferases DNMT3a/3b, but not with maintenance methyltransferase DNMT1. Thus, Kaiso may attract methyltransferases to surrounding regions and modulate genome methylation in renal cancer cells apart from being methyl DNA binding protein.

## 1. Introduction

DNA methylation is crucial for organism development, cell differentiation, X inactivation, genomic imprinting, and regulation of transcription. In vertebrates DNA methylation mainly occurs at CpG dinucleotides. Methylation of the promoter regions correlates with repression of transcription. About 70% of CpGs are methylated, except for those that are a part of so-called CpG islands. The establishment of DNA methylation occurs during the post-implantation period and changes during cell differentiation. In stem cells or differentiated cells, the pattern of DNA methylation is stably propagated over cell divisions. However, dynamic changes in DNA methylation may occur in nerve cells during learning and memory formation, in the aging period, during various pathological processes, after environmental impact [1,2,3,4].

Stability of methylation pattern is achieved by the equilibrium between maintenance of DNA methylation, the passive loss of methylation during replication, active demethylation, and de novo methylation. The establishment of DNA methylation is regulated by de novo DNA methyltransferases DNMT3a and DNMT3b, whereas active demethylation is performed by TET proteins via oxidation of methyl cytosines that are removed by base excision repair or during replication [5,6]. Although DNMT3a/b and TET enzymes have competitive activities in DNA methylation regulation, they appear to have a cooperative effect in maintaining the methylation pattern. For instance, TETs loss of function is associated with genome wide hypomethylation [7]. DNA methylation is initiated by a special histone marks pattern, by proteins that bind methylated DNA, by tissue-specific transcription factors [8]. Several studies have revealed many transcription factors that can interact with DNMT1 and DNMT3a/3b DNA methyltransferases [9,10]. Among proteins that interacted with DNA methyltransferases were found YY1, p53, MeCP2, MBD2, JUN, Sp1, and many others. It was shown previously that the deficiency of methyl-DNA binding protein Kaiso results in decreased methylation of ICR in *H19/Igf2* imprinted loci and *Oct4* promoter region [11,12]. Kaiso belongs to the BTB/POZ domain proteins family. It binds methylated DNA via three zinc fingers. Additionally, it can interact with sequences containing nonmethylated Kaiso binding site (KBS) CTGCNA, hydroxymethylation prevents its binding to DNA [13,14,15,16]. It was shown by X-ray diffraction data that the three-dimensional structure of Kaiso-mCpG DNA interaction is the same as KLF4-mCpG interaction [17]. KLF4 acts as a pioneering factor, it attracts TET2 dioxygenase resulting in DNA demethylation [18]. This suggests that these proteins may compete for binding to the methylated DNA and influence the DNA methylation profile. The main goal of this work is to discover if Kaiso regulates DNA methylation homeostasis.

Here, we show that deficiency in Kaiso causes mild genome wide hypermethylation but tends to keep some specific genic parts demethylated. Mechanistically we also asked whether Kaiso forms protein complexes with DNA methyltransferases.

## 2. Results

### 2.1. Kaiso (ZBTB33) Gene Editing

**Figure 1:**
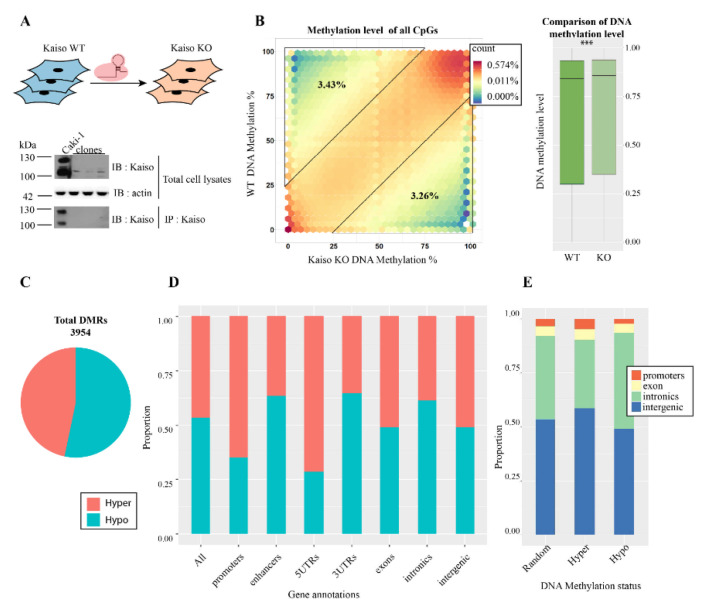
Whole-genome methylation in Caki1 cells and Kaiso deficient Caki1 cells. (**A**) Design of experiment, Kaiso was inactivated by CRISPR/Cas9 genome editing. (**B**) Hexagon plot showing methylation level for all CpGs (*n* = 28,368,572) in Kaiso deficient cells (Kaiso KO) versus wild type cells and mean methylation level over the whole-genome (*** *p*-value = 0.0002481). (**C**) Relative quantities of hyper- and hypo-DMRs. (**D**,**E**) Relative quantities of hypo- and hyper-DMRs that overlap with different genomic features.

We have used CRISPR/CAS9 genome editing system to introduce a frameshift into *Kaiso* (*ZBTB33*) gene in human clear renal carcinoma cells Caki1. This frameshift led to a stop in the BTB/POZ domain of Kaiso near 42 lysine (Figure 1A). Western blot analysis of total lysates confirmed the absence of Kaiso expression in Caki1 KaisoMut cells, although some non-specific bands can be observed. Additionally, immunoprecipitation with Kaiso antibodies confirmed that CRISPR/CAS9 editing was efficient (Figure 1A).

Next, we compared transcriptomes between Caki1 and Caki1 KaisoMut cells. RNA-seq analyses revealed that 442 genes were upregulated (padj < 0.001) and 260 were downregulated (Supplementary Table S1). GO (Gene Ontology) term analysis of upregulated mRNA revealed genes associated with RNA processing, metabolic processes, proteasome, and spliceosome functioning (Table S2). GO analysis of downregulated mRNA in the Kaiso deficient cell line identified transcripts associated with neurogenesis, ion binding, cell differentiation, and cell development (Table S2). The absence of Kaiso in Caki1 cells leads to a similar change in the gene transcription profile, which we observed early in HEK293 cells after analogous frameshift in the *Kaiso* gene [19] (Table S2). KEGG pathway analyses showed misregulation of genes involved in transcriptional misregulation in cancer, p53 signaling, ECM receptor interaction, MAPK signaling, spliceosome, and ubiquitin-mediated interaction (Table S2).

### 2.2. Kaiso Inactivation Causes an Increase of Genome Methylation

We performed whole-genome bisulfite sequencing in Caki1 KaisoMut cells to test if the Kaiso deficiency could change genome methylation level. Previously, we performed whole-genome bisulfite analysis for the Caki1 cell line [20,21]. Unexpectedly, we discovered a mild statistically significant increase of DNA methylation globally throughout the genome after Kaiso depletion (Figure 1B and Figure S1a). To find out whether this effect is associated with misregulation of the enzymes responsible for DNA methylation homeostasis we checked how their transcriptional level changes in Caki1 KaisoMut cells. Transcription of maintenance and de novo DNA methyltransferases (DNMT1, DNMT3a/3b), TET2, and TET3 dioxygenases remained stable in Kaiso deficient cells, while expression of TET1 dioxygenase was transcriptionally downregulated in Kaiso deficient cells (Figure S1b).

### 2.3. Kaiso Protects Promoters and CpG Islands against Hypermethylation and Enhancers from Hypomethylation

Methylome analysis showed that Kaiso deficiency resulted in the appearance of 3954 DMRs (differentially methylated regions): 2107 hypomethylated and 1847 hypermethylated (Figure 1C). Hypo- and hyper-methylated DMRs were found overall over the genome in intergenic, promoters, genic regions (Figure 1D). Hypermethylated DMRs were enriched at promoters and intergenic regions, while enrichment of hypomethylated DMRs was detected in introns. Comparative analysis of hyper- and hypo-methylated DMRs confirmed that in Kaiso deficient cells proportion of hypermethylated DMRs was higher in promoters, 5′UTR, and intergenic regions, while the opposite was observed in enhancers, 3′UTR, and introns (Figure 1E). Enhancers were taken from the FANTOM database [22], their activity was confirmed by the presence of the H3K27ac histone mark.

128 DMRs were found in CpG islands, from which 99 were hypermethylated. Outside the CpG islands there is a tendency to a decreased methylation (Figure S2a). Most hypermethylated CpG islands are located in intergenic regions.

**Figure 2:**
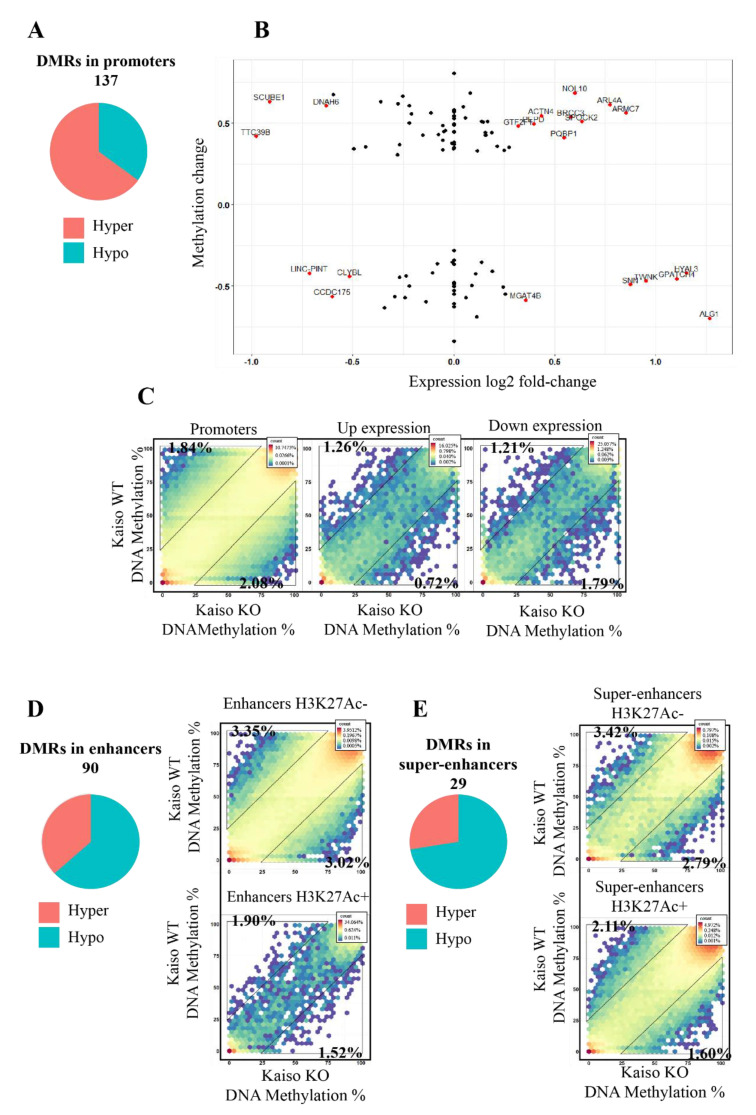
DMRs are associated with promoters and enhancers. Relative quantities of hyper- and hypo-DMRs that overlap with promoters (**A**), enhancers (**D**), and super-enhancers (**E**). (**B**) The relation between fold change of gene expression and the changes in DNA methylation within DMR in promoter regions. Hexagon plot showing methylation level for CpGs: (**C**) in all promoters (*n* = 1,514,345), in upregulated in Kaiso KO cells (*n* = 505,656), in downregulated in Kaiso KO cells (*n* = 32,328); (**D**) in enhancers (*n* = 254,090) with (*n* = 10,551) or without H3K27ac (*n* = 243,539); (**E**) in super-enhancers (*n* = 213,561) with (*n* = 162,969) or without H3K27ac (*n* = 50,592).

Among DMRs associated with Kaiso deficiency 137 intersect with promoters, 89 of which were hypermethylated and 48 DMRs hypomethylated (Figure 2A). Promoters hypermethylation was detected regardless of their activity (Figure S2b). Figure 2B shows what kind of changes in gene transcription happen in genes associated with DMRs in transcription start sites (TSS). We detected that most genes with DMRs in the promoter region are not expressed. The overall methylation level of CpGs in promoters tends to increase upon knockout of Kaiso (Figure 2C hexagon plot promoters, up-down). However, upregulated genes discovered more hypomethylated CpGs in Kaiso deficient cells than hypermethylated. It should be mentioned that many genes with DMRs in promoter regions have alternative transcription sites that may have an impact on transcription. DMRs in enhancers and super-enhancers in Kaiso deficient cells tend to be hypomethylated, regardless of their activity (Figure 2D,E).

### 2.4. ZBTB33, Nanog, and Oct4 Binding Sites Were Enriched within Hypomethylated CpGs in Kaiso Deficient Cells

**Figure 3:**
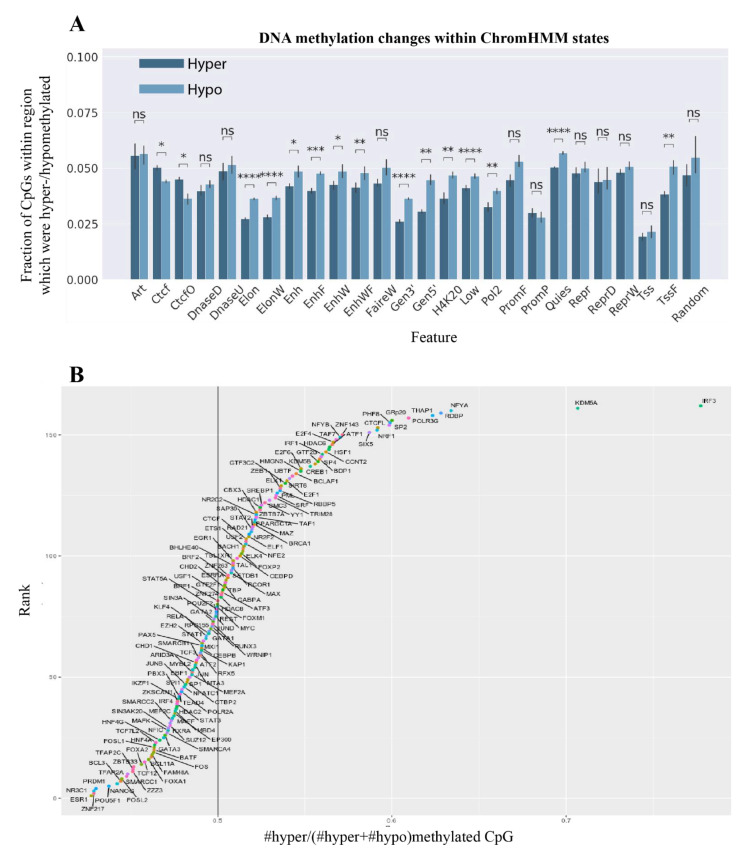
Distribution of hypo- and hyper-methylated CpGs within certain epigenomic features. (**A**) Region of ChromHMM genome subdividing (* *p*-value < 0.05, ** *p*-value < 0.01, *** *p*-value < 0.001, **** *p*-value < 0.0001, ns—not significant, Student’s *t*-test). (**B**) For indicated transcription factors we estimated the fraction of hypo- and hypermethylated sites among all CpGs with significantly altered methylation.

We explored DNA methylation changes within various genomic and epigenomic layouts. We used genome segmentation performed by ChromHMM [23]. ChromHMM binned the genome according to the data on multiple genomic features into non-overlapped parts representing different chromatin states. Although weak hypermethylation in Kaiso deficient cells is observed at a genome wide level, analysis of regions with different chromatin states showed mostly hypomethylated regions (Figure 3A). Only one type of sequence is hypermethylated, these are CTCF occupied sites DNAse I sensitive (CTCFO) or not (CTCF). CTCF occupied sites often resemble to insulators and cis-regulatory modules. Hypomethylated CpGs were detected in parts involved in transcriptional elongation, genic regions, transcriptional termination regions (Elon, ElonW, Pol2, H4K20me1), enhancers, and their environment (Enh, EnhW, TSSF). Interestingly, among hypomethylated regions in Kaiso deficient states, we observed regions devoid of histone modifications with (Low) or without (Quies) transcriptional activity. The Quies states showed a consistent enrichment for the nuclear lamina domains and are transcribed infrequently [24]. Promoter regions show a rather unchanged DNA methylation pattern with equal levels of hypo- and hyper-methylated CpGs in Kaiso deficient cells.

Next, we discover what happened with methylation of various transcription factor binding sites. We used a set of transcription factor binding sites annotated by ENCODE. We found both categories of binding sites: hyper- and hypo-methylated (Figure 3B). Among CpG sites with significant hypomethylation in Kaiso deficient cells, we found Kaiso’s binding regions (ZBTB33), Znf217 oncogene sites, pluripotent factors sites (Oct4 and Nanog), estrogen receptor binding sites and so on. Among hypermethylated sites, we again found CTCF binding regions, KDM5a histone H3k4me3 demethylase, IRF3 and others. To discover how the changes of DNA methylation in binding regions of transcription factors (TF) corresponded to gene transcription changes in Kaiso deficient cells, we analyzed the expression of these transcription factors. We detected a set of differentially expressed TF with altered methylation in their binding sites (Figure S3). Among overexpressed TF whose binding sites were hypermethylated, we observed a whole set of transcriptional repressors (HDAC1, ZBTB7a, Max, E2F4, SRF, Elk1, ZNF263, TRIM28). While for transcription repressors Esr1, TFAP2AD, Bcl3 with a decreased expression we detected hypomethylation of their binding sites. Many of these repressors recruit various corepressor complexes containing DNA methyltransferases, they play a key role in the establishment of DNA methylation pattern of target sequences [25,26,27]. So, the changes in the expression of transcriptional repressors maybe involved in hypermethylation in Kaiso deficient cells.

### 2.5. Hypomethylation of TRIM25 Promoter in Kaiso Deficient Cells Is Reversible

**Figure 4:**
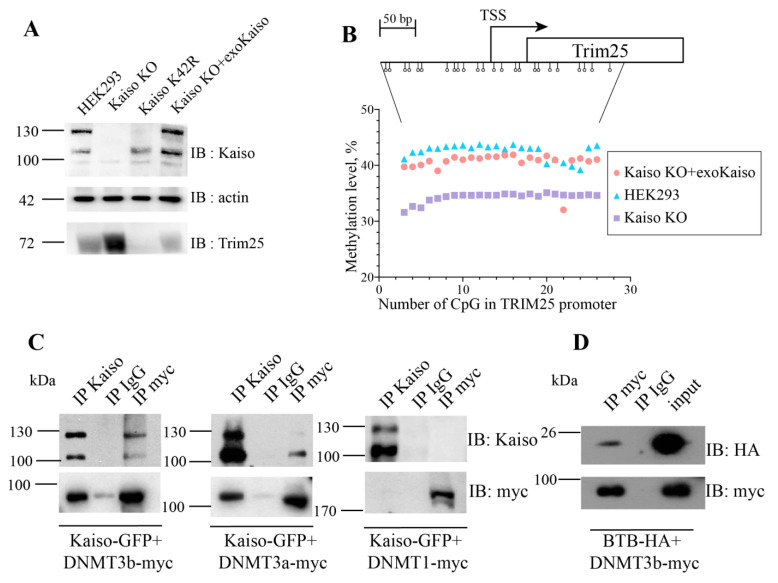
Kaiso regulates *TRIM25* promoter methylation. (**A**) Western blot analyses of cell lysates from HEK293 cells, Kaiso deficient (Kaiso KO) and K42R mutant cells for expression of TRIM25. (**B**) Analysis of promoter *TRIM25* methylation by bisulfite conversion followed by NGS sequencing. Open circles mean CpG dinucleotides. (**C**) Kaiso-GFP was cotransfected with DNMT1-myc, DNMT3a-myc, or DNMT3b-myc Western blot analysis after co-immunoprecipitation. (**D**) BTB-HA was cotransfected with DNMT3b-myc. Western blot analysis of co-immunoprecipitation.

Since ChromHMM showed that hypomethylated regions are more common, we decided to test whether hypomethylation would be reversible. As a model system, we chose a promoter of *TRIM25* gene. Earlier, we demonstrated that Kaiso is involved in the regulation of the *TRIM25* promoter activity. Kaiso binds to *TRIM25* promoter in chromatin immunoprecipitation experiments. Its deficiency led to TRIM25 upregulation, while mutation on 42 lysine in BTB/POZ domain of Kaiso resulted in repression of TRIM25 [19]. To confirm that the change in *TRIM25* transcription is associated with a change in the methylation level of its promoter, we carried out a bisulfite analysis. Genomic DNA from wild-type cells, Kaiso deficient cells, and cells expressing exogenous Kaiso was converted by bisulfite. After the bisulfite conversion region corresponding to the *TRIM25* promoter was amplified and analyzed by NGS sequencing. In wild-type cells, the level of *TRIM25* promoter methylation was about 40% (Figure 4A,B). Kaiso deficiency results in slightly decreased of methylation level to 30%. Reduction of *TRIM25* promoter methylation was following increased transcription of *TRIM25* in Kaiso KO cells. The reduction of methylation was reverted by expression of exogenous Kaiso as it was shown by bisulfite analysis (Figure 4B). Recovery of Kaiso expression by lentivirus transfection resulted in TRIM25 downregulation according to increasing promoter methylation. So, Kaiso is involved in the methylation maintenance of this region.

### 2.6. Kaiso Formed a Complex with De Novo DNA Methyltransferases

Since Kaiso can increase the methylation level of the *TRIM25* promoter, we assumed that Kaiso might attract DNA methyltransferases. To check whether Kaiso may form a complex with DNA methyltransferases, we cotransfected Kaiso-GFP and myc tagged DNMT1, DNMT3a, or DNMT3b into HEK293 cells. Co-immunoprecipitation with myc antibodies revealed that Kaiso forms a complex with DNMT3a/3b, but not with DNMT1 (Figure 4C). To confirm this complex formation, we performed immunoprecipitation with antibodies against Kaiso. Western blot analyses demonstrated that Kaiso forms a complex with de novo DNA methyltransferases. Further, we determined which domains of Kaiso are essential for complex formation. Kaiso consists of BTB/POZ domain and C-termini zinc finger domain. BTB/POZ domain is responsible for protein-protein interaction. Therefore, we investigated how important is the BTB/POZ domain of Kaiso for interaction with DNMT3b. We cotransfected BTB domain tagged with HA along with DNMT3b-myc and immunoprecipitated proteins with myc antibodies. Western blot analyses showed that the BTB/POZ domain of Kaiso is sufficient for complex formation with de novo DNA methyltransferase DNMT3b (Figure 4D).

Then, we investigated whether de novo DNA methyltransferase directly interacts with Kaiso, or if they just formed a multisubunit complex with each other. To resolve this question, we performed a co-precipitation assay. Full length Kaiso was tagged with GST and produced in the *E. coli* expression system. Empty GST was used as a control. We performed pull-down with total cell lysates from HEK293 transfected with DNMT3b-myc. Western blot analyses of precipitated proteins revealed that DNMT3b and Kaiso could not interact directly with each other (Figure S4). Consequently, they can exist in one complex, but they do not interact directly.

### 2.7. Inactivation of KLF4 Abolished Kaiso Dependent Demethylation of TRIM25 Promoter

**Figure 5:**
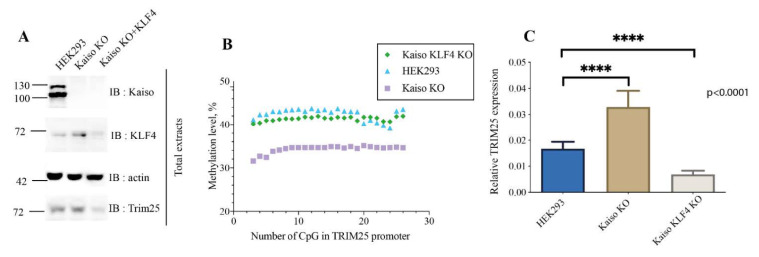
KLF4 is important for Kaiso-dependent regulation of *TRIM25* promoter methylation. (**A**) Western blot analyses of total cell lysates from HEK293 cells, Kaiso KO, double knockout (KLF4 KO and Kaiso KO) mutant cell lines. (**B**) Analysis of promoter *TRIM25* methylation by bisulfite conversion followed by NGS sequencing in HEK293, Kaiso KO and double knockout (Kaiso, KLF4). (**C**) qRT-PCR analysis with Taqman probe for *TRIM25* transcription relative to GAPDH expression level (*n* = 3, **** *p*-value < 0.0001, Student’s *t*-test).

Finally, we asked whether KLF4, which attracts TET enzymes to demethylate DNA, may compete with Kaiso for binding sites. KLF4 binds the same methylated sites as Kaiso does. To answer this question, we deleted KLF4 along with Kaiso by frameshift via CRISPR/CAS9 editing (Figure 5A). The absence of Kaiso and KLF4 expression was confirmed by Sanger sequencing and Western blot analyses. Bisulfite analyses of *TRIM25* promoter showed that in the absence of KLF4 deficiency of Kaiso did not change the methylation level of *TRIM25* (Figure 5B). Interestingly, that TRIM25 expression level was decreased, as it was shown by Real time PCR and Western blot analyses (Figure 5A,C). So, *TRIM25* promoter demethylation is dependent on the presence of KLF4.

## 3. Discussion

In this work, we have determined how Kaiso influences DNA methylation in human kidney cancer cells. Until now, it was believed that Kaiso is a transcriptional repressor or transcriptional activator, recruiting various corepressor complexes. It is involved in the oncogenic transformation of cells. Kaiso knockout leads to the delayed formation of smaller tumors in the intestine in *APC* ^min/−^ cancer model systems [28]. An increase of Kaiso expression, on the contrary, promotes an active inflammatory process, which leads to a more pronounced formation and progression of intestinal tumors [29]. In human breast cancer, the expression of Kaiso is one of the poor prognostic markers [30,31]. One of the Kaiso functions that are associated with cell malignancy is based on the regulation of oncogenes transcription: *MTA2*, *MMP7*, *CCND1* [32]. For the first time, we determined that Kaiso influences the DNA methylation in human kidney cancer cells, which regions change their methylation upon knockout of the Kaiso gene, that Kaiso can co-immunoprecipitate with de novo DNA methyltransferases.

In the absence of Kaiso expression, a mild increase in CpGs methylation is observed, which is detected throughout the genome and is especially noticeable in the regions that can bind to the insulator protein CTCF. Such a uniform distribution of hypermethylated CpGs across the genome suggests that this may be a nonspecific effect, which is possibly associated with a decrease in *TET1* dioxygenase transcription in Kaiso-deficient cells. TET1 resembles for oxidation of 5-methylcytosine to 5-hydroxymethylcytosine, 5-formylcytosine, 5-carboxycytosine, which are recognized by a DNA glycosylase and exposed to a based excision repair leading to demethylation. Thus, a decrease in the level of TET1 may lead to a decrease in demethylation processes, and therefore leads to DNA hypermethylation.

It was previously shown that CTCF and Kaiso occupied different alleles in the differentially methylated region of *H19/Igf2* locus: Kaiso was detected on the methylated allele, while CTCF was found on the unmethylated one [33,34]. So, they are physically located on different alleles and cannot interact with each other. However, later research revealed that Kaiso interacts with CTCF in the yeast two-hybrid system and co-immunoprecipitated in lysates from HeLa cells [35]. Here, we observed increased methylation of CTCF binding sites in Kaiso deficient cells. Hypermethylation of CTCF binding sites was detected in cells with CTCF loss [36]. However, in Kaiso deficient cells *CTCF* is highly expressed as it was shown by RNA-seq analyses. So, we assume that Kaiso can attract CTCF to DNA, which will keep DNA in a hypomethylated state. In order to resolve this issue in the future, it is necessary to determine the binding sites of CTCF and Kaiso in the obtained cell lines.

Despite an increase in genome wide methylation, we found more hypomethylated DMRs than hypermethylated ones. This result reconfirms that the distribution of hypermethylated CpGs is more uniform across the genome than the distribution of hypomethylated CpGs. ChromHMM analysis showed that hypomethylated CpGs are predominantly located in gene bodies, enhancers, and regions that do not contain histone marks and are actively transcribed or remained inactive. These inactive regions without modifications of histones may resemble the nuclear lamina domains that are responsible for targeting genomic sites to the nuclear periphery [37]. We also observe hypomethylation of repetitive regions in Kaiso-deficient cells, which, in turn, can lead to genome instability. For some cancer conditions genomic repeat instability is a driving factor [38]. Along with hypomethylation of enhancers in Kaiso deficient cells, we detected demethylation of super-enhancers and binding sites for Kaiso, for pluripotent factors Nanog and Oct4. So, Kaiso protects genic regions, enhancers, and regions without histone modifications from demethylation. Further, it would be interesting to determine the Kaiso binding sites in the Caki1 cell line. This would make it possible to determine whether the changes in DNA methylation upon knockout of Kaiso are related to its binding sites. Now we can assume that protection from hypomethylation is explained by the ability of Kaiso to interact with DNMT3a/3b methyltransferases. We showed that Kaiso is found in one complex with de novo DNA methyltransferases, but not with DNMT1. Using *TRIM25* promoter as a model system we confirmed that Kaiso is important for *TRIM25* promoter status methylation: deficiency of Kaiso led to promoter demethylation, while recovery of Kaiso expression results in increased methylation. In the analysis of whole genome methylation data in Caki1 Kaiso deficient cells we also detected hypomethylation of potential Kaiso binding regions. So, Kaiso protects its binding sites from demethylation.

Interestingly, DNA methylation in cancer cells is decreased in comparison with healthy tissues. Demethylation especially affects genic regions, enhancers, and binding sites for pluripotent factor Nanog [39]. Hypomethylation of binding sites for stemness- and proliferation-associated transcription factors (Oct4, Nanog, Sox2) was detected in circulating tumor cells that formed clusters linked to increased metastatic potential [40]. So, on one hand, DNA hypermethylation in Kaiso deficient cells may be related to changes in tumor aggressiveness, as it was observed for triple-negative breast cancers cell lines. In these cells Kaiso deletion attenuates proliferation, leading to delayed tumor onset in mice xenografted [30]. On the other hand, it may change cell metastatic potential. Further investigations need to clarify the role of Kaiso in tumorigenesis.

During the first days of somatic cells reprogramming enhancers and especially super-enhancers are demethylated [41]. Previously we demonstrated that Kaiso gene knockout in mouse embryonic fibroblasts results in accelerated and more efficient reprogramming [12]. This effect may be also related to decreased methylation of enhancers and super-enhancers in the Kaiso knockout cells. Moreover, demethylation of enhancers and SE during reprogramming is closely related to KLF4 binding, which can attract TET2 dioxygenase [18]. We demonstrated that in the absence of the KLF4, Kaiso knockout cannot change *TRIM25* promoter methylation, while a mutation in Kaiso only led to *TRIM25* promoter demethylation. So, Kaiso may regulate DNA methylation homeostasis in different ways: attracting de novo DNA methyltransferases, competing with KLF4 for binding to target sites, and influencing transcription of *TET1* dioxygenase.

Thus, we have shown for the first time that Kaiso can affect the total genome methylation level by protecting enhancers, super-enhancers, Oct4, and Nanog binding sites from hypermethylation.

## 4. Materials and Methods

### 4.1. Cell Lines

Caki-1, HEK293, HEK293T, Kaiso KO, and K42R Kaiso HEK293 cells [19] were grown in Dulbecco’s modified Eagle medium supplemented with 10% fetal bovine serum, 1% penicillin/streptomycin, and 2 mm l-glutamine. Cells were transfected with Lipofectamine 2000 (Thermo Fisher Scientific, Waltham, MA, USA) according to the manufacturer’s protocol. Cells were typically harvested 48 h post-transfection for further analysis.

### 4.2. Plasmids and Vectors

Kaiso-GFP, BTB-HA, pX459-Kaiso constructs were used the same as in [19]. Full-length Kaiso (1–692) was cloned in pGex-2T. pcDNA3/Myc-DNMT3B1 (Addgene plasmid # 35522), pcDNA3/Myc-DNMT3A (Addgene plasmid # 35521), pcDNA3/Myc-DNMT1 (Addgene plasmid # 36939) were a gifts from Arthur Riggs [42,43]. For lentivirus purification, we used the pCDF system (System Bioscience): pVSV-G pFiv-34N plasmids. Kaiso was subcloned into pCDF1lentivirus-MCS2-EF1-Puro vector.

Plasmid for *KLF4* editing was obtained as follows: ligation of BbsI-digested pSPCas9(BB)-2A-Puro (PX459) (Addgene # 48139) plasmid with annealed sgRNA oligo insert: Crisp_KLF4_for 5′-CACCGGAGCCGGTGCGGCTTGCGG, Crisp_KLF4_rev 5′-AAACCCGCAAGCCGCACCGGCTCC.All plasmids were verified by Sanger sequence analysis.

### 4.3. qRT-PCR

RNA was extracted using Trizol (Thermo Fisher Scientific) according to manufacturer instructions. Total RNA (2 μg) was treated with 2 U DNase I (Thermo Fisher Scientific) and reverse transcribed from random hexamers and Revertaid First Strand cDNA synthesis kit (Thermo Fisher Scientific). qRT-PCR was performed using TaqMan probes on the CFX96 (BioRad) instrument. Amplification of *GAPDH* transcript served as RNA controls for relative quantitation. Used primers: *TRIM25* (for: 5′-GCCTGGTGGAGCATAAGACC-3′; rev: 5′-TCTGACTGTACATGACAGTTAGT-3′; probe: FAM 5′-CCTGGAGGCCACCCTGAGGCAC-3′ RTQ1), *GAPDH* (for: 5′-ACCAGGGCTGCTTTTAACTC-3′; rev: 5′-AGATGGTGATGGGATTTCCA; probe: FAM 5′-TCATTGACCTCAACTACATGGTTTACA-3 RTQ1).

### 4.4. Genome Editing

CRISPR/CAS9–based editing was performed as described in ref. [44]. Caki1 cells were transfected with pX459-Kaiso plasmid, HEK293 cells were transfected with the pX459-KLF4 or pX459-Kaiso or with both plasmids to generate frameshift. Puromycin was applied after 24 h at a concentration of 1 μg/mL for HEK293 cells and 2 μg/mL for Caki1 cells. Cells were incubated for 72 h, passaged into a 96-well plate at a density of 1 cell per 2 wells on a medium without puromycin. The frameshift in the *KLF4* gene was confirmed by Sanger sequencing of the corresponding amplicons obtained from PCR with genomic DNA (KLF4_for 5′-TCCCACATGAAGCGACTTCC, KLF4_rev 5′-GGATGGGTCAGCGAATTGGA; Kaiso_for 5′-CATGGAGAGTAGAAAACTGATTT, Kaiso_rev 5′-CACTCCTAATAACTGCCCTGA).

### 4.5. Lentiviral Transduction

For virus production HEK293T cells were transfected with pCDF-Kaiso, pVSV-G, and pFiv-34N vectors. Viral supernatant was collected after 48 and 60 h, filtered through a 45 µm filter. The virus was precipitated by 12.5% PEG 8000 at +4 °C overnight followed by centrifugation at 4200× *g* during 40 min at +4 °C. Pellet was resuspended in OPTI-MEM and stored at −80 °C. Infections were carried out in HEK293 cells in a 60-mm cell plate for 12 h with addition of polybrene Infected cells were selected by puromycin (final concentration 1 µg/mL) for 3 days. Then cells were cultivated without puromycin.

### 4.6. Protein Expression and Purification

GST-containing proteins were expressed in *E. coli* BL21 cells induced by the addition of IPTG to final concentration 0.1 mM for 3 h at 20 °C. After cell sonication proteins were affinity purified using a glutathione sepharose 4b and remained on sepharose for pull-down (GE Healthcare, Chicago, IL, USA). 

### 4.7. GST-Coprecipitation

Glutathione sepharose 4B-conjugated Kaiso and empty GST (≈20 μg) were incubated with RIPA total cell lysates for 3 h at 4 °C, washed 4 times with buffer (10 mM Tris pH 7.4, 1 mM EDTA, 1 mM EGTA, 150 mM NaCl, 1% Triton X-100) and eluted by addition of SDS-loading buffer.

### 4.8. RNA-Seq

Total RNA was extracted from cells with TRIzol reagent according to the manufacturer’s instructions (Thermo Fisher Scientific) in triplicate. Quality was checked with BioAnalyser and RNA 6000 Nano Kit (Agilent Technologies, Santa Clara, CA, USA). PolyA RNA was purified with Dynabeads^®^ mRNA Purification Kit (Ambion, Inc., Austin, TX, USA). An Illumina library was made from polyA RNA with NEBNext^®^ Ultra™ II RNA Library Prep Kit for Illumina^®^ (NEB) according to the manual. Sequencing was performed on HiSeq1500 with a 50 bp read length (BioProject ID PRJNA386575). At least 10 million reads were generated for each sample. The RNA reads were filtered by quality (phred > 20) and library adapters were trimmed using Cutadapt software (version 1.12) (Marcel, 2011). Afterward the reads were mapped to the human genome (GRCh37/hg19) by STAR [45]. Gene models of non-overlapping exonic fragments were taken from the RefSeq database. For each gene, we found total coverage by mapped reads in each sample with bedtools coverage (version 2.26.0). And gene expression quantified using HTSEQ v0.11.1 [46] Differential expression analysis was performed by applying default read count normalization (estimateSizeFactors) and performing per-gene negative binomial tests (nbinomTest), implemented in DESeq2 package (Love MI, 2014), with three replicates in each group with default parameters 15. Enriched Gene Ontology terms were identified using DAVID bioinformatics resources with genes pre-ranked according to their fold change induced by deficiency of Kaiso [47].

### 4.9. ChIP-Seq

Chromatin immunoprecipitation was performed with a magnetic kit (ab156907) according to the manufacturer’s instructions. Immunoprecipitation was performed with H3K27ac antibodies in duplicate. Libraries were constructed with NEBNext^®^ Ultra™ II DNA Library Prep Kit for Illumina^®^ (NEB) and sequenced with a single read mode of 50 bp read length. Read amount was adjusted to reach peak saturation. For quality control of DNA sequencing data, we used FastQC (Andrews S. 2010) software. Reads with quality less than 20 were filtered using Cutadapt and were also truncated reads to 50 nucleotides. The remaining reads were mapped to the human genome (GRCh37/hg19) using a Bowtie2 base with the ‘end-to-end’ option. MACS2 [48], were used to perform the peak calling and the peaks were annotated using Bedtools.

### 4.10. Bisulfite Conversion and Whole-Genome Bisulfite Sequencing

DNA methylation was profiled by whole-genome bisulfite sequencing (WGBS) was performed as reported previously [21]. Briefly, two micrograms of genomic DNA and 4 ng of lambda phage DNA were sonicated to an average size of 250 bp with Covaris S2. Libraries were constructed with NEBNext^®^ Ultra™ II DNA Library Prep Kit for Illumina^®^ (NEB) and adapters with cytosine replaced to methylcytosine. Two biological replicates from the Caki1 cell line Kaisomut were taken for bisulfite sequencing.

### 4.11. DNA Methylation Analyses

Reads were aligned to GRCh37/hg19 genome assembly with *Bismark* software [49]. Bisulfite conversion efficiency (>99%) was assessed using both lambda phage and methylation of non-CpG context. Individual differential CpGs were identified using the beta-binomial regression approach implemented in the *MethPipe* pipeline [50]. To obtain differentially methylated regions (DMRs) we first performed a Chi-squared test on individual CpGs which were covered at least by four reads in each replicate, then we carried out Benjamini-Hochberg adjustment. Consecutive CpGs which fall under the criteria (FDR < 0.2, methylation difference > 0.1) were defined as a DMR. The contingency table for the Chi-squared test consisted of methylated and unmethylated read numbers as was described in [51].

### 4.12. ChromHMM Analysis

The following tracks were loaded from the ENCODE database: wgEncodeAwgSegmentationChromhmm*, wgEncodeRegTfbsClusteredV3. For all Chromhmm states, we have estimated a ratio of hyper- or hypomethylated CpGs to all CpGs. Similarly, conserved TF binding sites were taken from the TFbs track. The proportion #Hyper/(#Hyper+#Hypo) was calculated for each transcription factor.

### 4.13. Antibodies

The following reagents were used in this study: anti-Kaiso polyclonal rabbit antibodies (kindly gifted by Dr. A. Reynolds), anti-HA (H6908, Sigma-Aldrich, St. Louis, MI, USA), anti-HA agarose (A2095, Sigma-Aldrich), anti-actin (ab8227, Abcam, Cambridge, UK), anti-Kaiso 6F (ab12723, Sigma-Aldrich), anti-myc (ab9106, Abcam), anti-myc mouse (kindly gifter by Dr. I. Deyev [52]), anti-H3K27ac (ab4729, Abcam), anti-KLF4 (ab215036, Abcam), anti-TRIM25 (ab86365, Abcam).

### 4.14. Target DNA Methylation Analyses

Genomic DNA was extracted with DNeasy Blood and Tissue Kits (QIAGEN, Hilden, Germany). DNA was subjected to bisulfite conversion using EZ DNA Methylation Kit (Zymo Research) and amplified using primers corresponding TRIM25 promoter region for 5′-TTGAATTCTTAGATGAGTGTTGGGAAGG.

Rev 5′-TTGGATCCAATCGAAACACAACTACTACACC. PCR product was used as a DNA template to make Illumina compatible library with NEB E7645S kit according to the manual. The library was sequenced on HiSeq 1500 in SR mode with 250 bp read length.

### 4.15. Immunoprecipitation Analyses

Immunoprecipitation was performed using anti-Kaiso 6F, anti-myc mouse, control IgG antibodies, and HA-agarose. Lysates of HEK293 cells or transfected cells were used. HEK293 cells were seeded in a 6-well plate and transfected with Lipofectamine 3000 reagent (Thermo Fisher Scientific) according to the manufacturer’s instructions. After 2 days, cells were washed with 1× PBS and lysed in 300 μL of cold RIPA buffer with protease inhibitor (Roche) and 10 mM NEM for 10 min on ice. Lysates were centrifuged at 16,000 rpm and +4 °C for 10 min. The supernatants were stored at −70 °C. To the supernatants, 1 μg of anti-Kaiso (6F), HA, TRIM28, myc, or control IgG antibodies were added and incubated at 4 °C with rotation overnight. Thereafter, 20 μL of G-Sepharose protein (Amersham) was added and incubated with rotation. Then washed three times with wash buffer (10 mM Tris-HCl pH 7.4, 1 mM EDTA, 1 mM EGTA, 150 mM NaCl, 1% Triton x100) and added to the mixture of supernatant and antibodies. The proteins were eluted by adding 60 μL of SDS loading buffer for electrophoresis and heating to 95 °C for 5 min. The supernatant was used for electrophoresis followed by Western blotting.

## Data Availability

All RNA-seq and WGBS raw data have been submitted to the Sequence Read Archive under the accession number PRJNA734133.

## Supplementary Materials

The following are available online at https://www.mdpi.com/article/10.3390/ijms22147587/s1, Figure S1: Genome wide changes in DNA methylation upon Kaiso knockout, Figure S2: CpG island and promoters preferentially hypermethylated in Kaiso deficent cells; Figure S3: The changes of DNA methylation in binding sites of transcription factors; Figure S4: In coprecipitation DNMT3b and Kaiso can not interact directly with each other. Table S1: List of genes up and down regulated in Caki1 Kaiso deficient cells; Table S2: GO analyses of differentially expressed genes.
