# Supplementary material for: Kaiso Regulates DNA Methylation Homeostasis
Source: Int J Mol Sci. 2021 Jul 15;22(14):7587. doi: 10.3390/ijms22147587 (PMC8307659; doi:10.3390/ijms22147587)
Supplement: Supplementary file 1 [file ijms-22-07587-s001.zip › Supplementary copy.pdf]

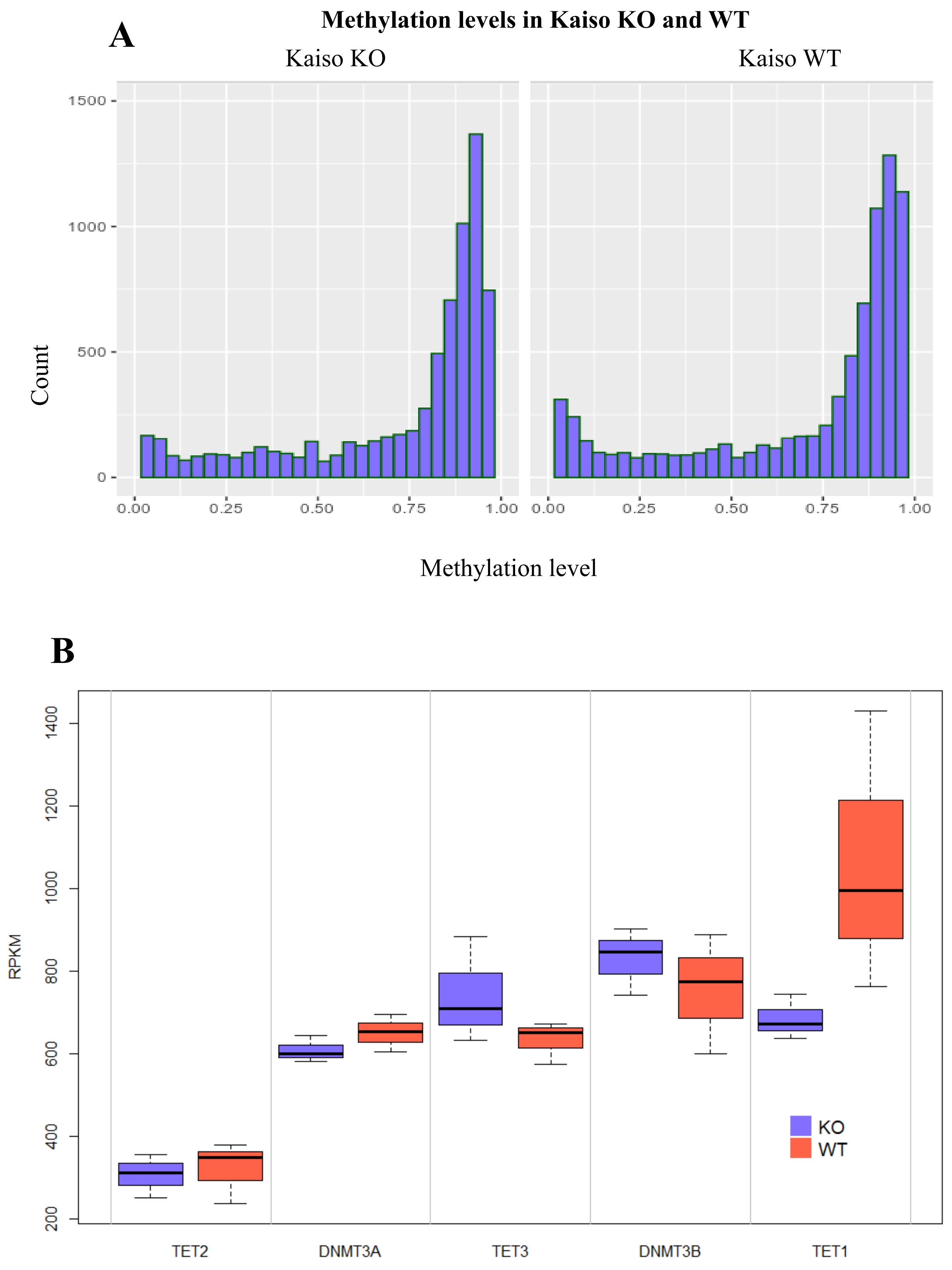

Supplementary figure 1. Genome wide changes in DNA methylation upon Kaiso knockout A, The distribution of DNA methylation levels in the Kaiso-deficient and wildtype cells

B, Gene expression of TET1, TET2, TET3 dioxegenases and DNMT1, DNMT3a and DNMT3b DNA methyltransferases

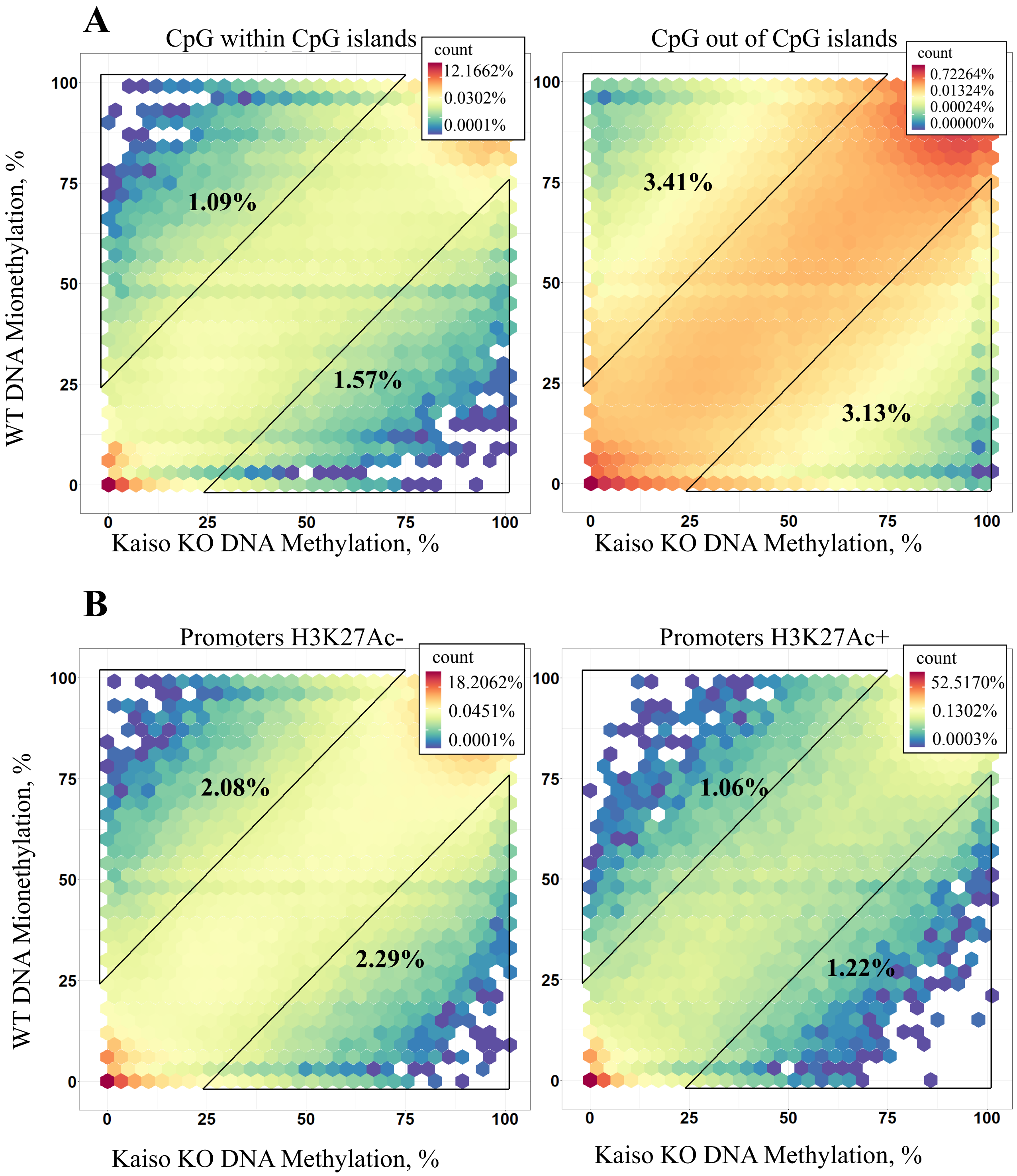

Supplementary figure 2. CpG island and promoters preferentially hypermethylated in Kaiso deficent cells. Hexagon plots showing methylation levelfor all CpGs within and out CPG islands (**A**), within H3k27ac positive and negative regions (**B**)

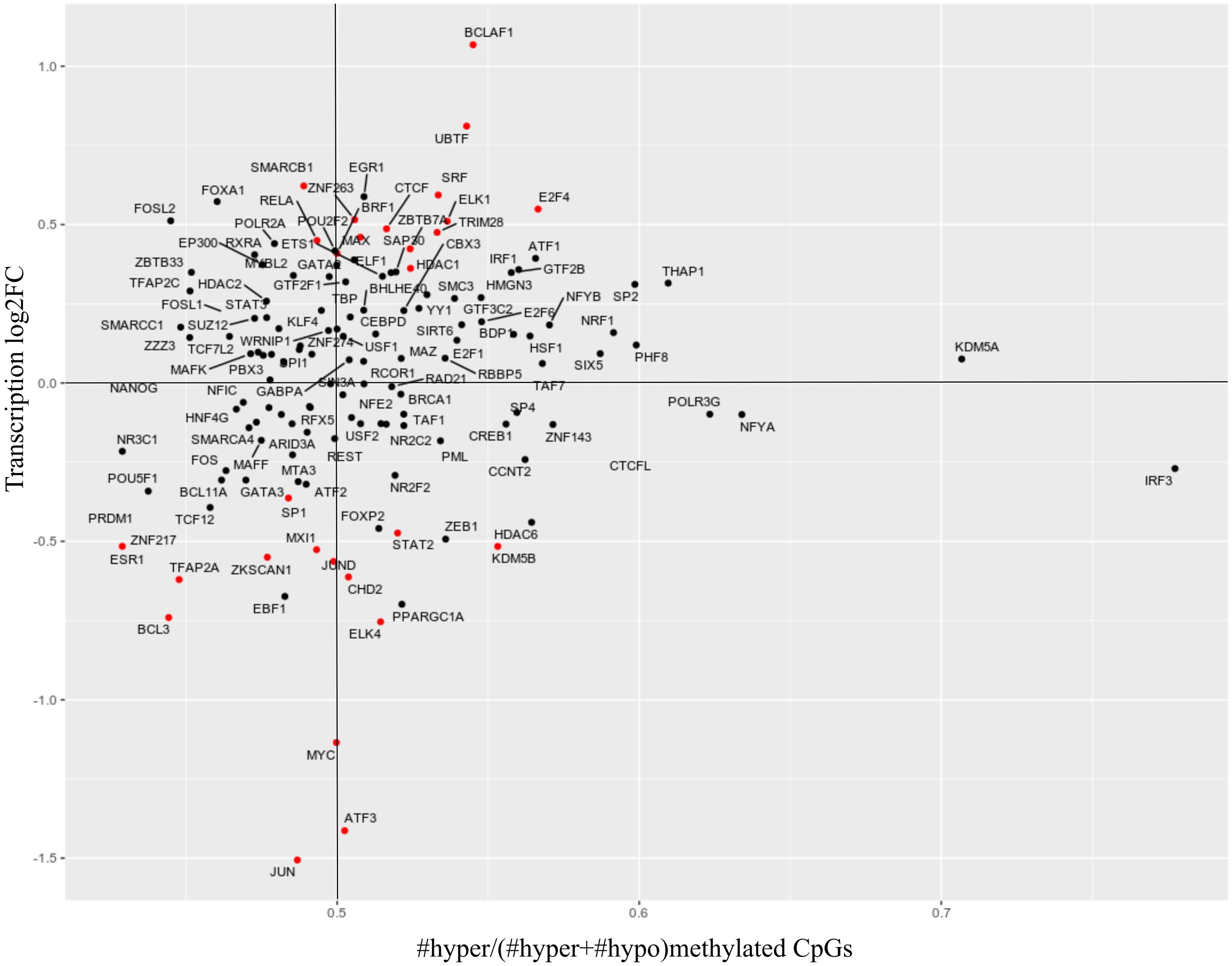

Supplementary figure 3. The changes of DNA methylation in binding sites of transcription factors corresponded to gene transcription changes of these transcription factors in Kaiso deficient cells.

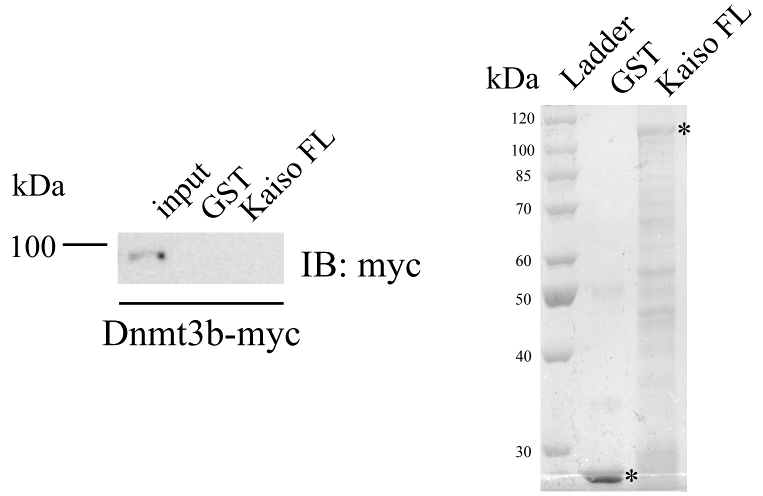

Supplementary figure 4. In coprecipitation DNMT3b and Kaiso can not interact directly with each other. Western blot with GST-pull down (left) and coomassie gel staining of Kaiso FL fused with GST(right).
